# Novel synthesized 2, 4-DAPG analogues: antifungal activity, mechanism and toxicology

**DOI:** 10.1038/srep32266

**Published:** 2016-08-26

**Authors:** Liang Gong, Haibo Tan, Feng Chen, Taotao Li, Jianyu Zhu, Qijie Jian, Debao Yuan, Liangxiong Xu, Wenzhong Hu, Yueming Jiang, Xuewu Duan

**Affiliations:** 1 Key Laboratory of Plant Resource Conservation and Sustainable Utilization/Guangdong Provincial Key Laboratory of Applied Botany, South China Botanical Garden, Chinese Academy of Sciences, Guangzhou 510650, Guangdong Province, China; 2 Department of Food, Nutrition and Packaging Sciences, Clemson University,Clemson, South Carolina, USA; 3 School of Minerals Processing and Bioengineering, Central South University, Changsha 410083, China; 4 Department of Food Engineering, College of Life Science, Dalian NationalitiesUniversity, Dalian 116600, China

## Abstract

2, 4-Diacetylphloroglucinol (2,4-DAPG), a natural phenolic compound, has been investigated in light of its biological activities against plant pathogens. To improve its potential application, fourteen 2,4-DAPG analogous were synthesized through the Friedel-Crafts reaction using acyl chlorides and phloroglucinol. Of the 2,4-DAPG derivatives, **MP4** exhibited much higher antifungal activity against *Penicillium digitatum* and *P. italicum*, the major pathogenic fungi in citrus fruit, than 2, 4-DAPG in *vitro*, and significantly inhibited the development of decay in harvested mandarin (*Citrus reticulata* Blanco cv. Shatang.) fruit *in vivo*. It was found that MP4 resulted in the wrinkle of the hyphae in both fungi with serious folds and breakage. In addition, the expression of several cytochrome P450 (*CYP*) genes were also modified in both fungi by MP4, which might be associated with the disorder of cell membrane formation. Furthermore, the toxicology of **MP4** by evaluating the cell proliferation effect on human normal lung epithelial (16HBE) and kidney 293 (HEK293) cells, was significantly lower than that of albesilate, a widely used fungicide in harvested citrus fruit. In summary, the synthesized **MP4** has shown a great potential as a novel fungicide that might be useful for control of postharvest decay in citrus fruit.

Citrus is one of the most economically important fruits with rich amounts of nutrients, but it is highly susceptible to plant pathogens mainly including *P.digitatum* and *P. italicum*. Generally speaking, these two pathogens account for approximately 15–50% of annual economic loss of the total amount of citrus production worldwide^1^. Currently, chemical fungicides, such as iminoctadinetris (albesilate), thiabendazole (TBZ) and imazalil (IMZ), have been widely used to minimize the postharvest decay of citrus caused by *P. digitatum* and *P. italicum*. However, extensive application of fungicides has caused much attention in concern to severe resistance of *P. digitatum* and *P. italicum* after their long-term exposure to the fungicides^2^,^3^. For instance, it has been reported that the resistant frequency of the *P. digitatum* isolates to IMZ increased 28.5–40 folds in 2005–2010 compared with those in 2000 in Zhejiang, China^4^. Therefore, there is an urgent requirement to search alternate chemicals that can be used for post-harvest handling of citrus.

On the other hand, novel fungicides should undergo toxicity evaluations prior to their practical applications because fungicides are normally associated with secondary effects on non-target species, including human beings, through the food chain. As a result, risk assessment is particularly essential for novel chemicals with the potential to be used as fungicides^5^, of which the toxicology is a study of adverse effects of the chemicals on living systems including cells, tissues, or organisms. For examples, Caron-Beaudoin *et al.*^6^ reported the adverse effects of atrazine on human adrenocortical carcinoma (H295R) and primary umbilical vein endothelial (HUVEC) cells^6^. Abhishek *et al.*^7^ reported the toxicity of three pesticides (i.e., alpha-hexachlorocyclohexane, parathion methyl and carbofuran) on human keratinocyte cells^7^.

2, 4-Diacetylphloroglucinol (2, 4-DAPG), which is one of the most intriguing compounds produced by some strains of the bacterium *Pseudomonas* fluorescens^8^,^9^, was originally paid attention in light of its antimicrobial properties. Later it was found to be a potent fungicide against various plant pathogens, such as *Gaeumannomy cesgraminis* var. tritici^10^,^11^. Since structural modification of natural products is one of the most attractive research fields for the development of new drugs, it is reasonable to develop novel 2,4-DAPG analogues in order to improve its potential biological activities, and to expand its antifungal spectrum.

It is well recognized that biophysical processes play important roles in the antimicrobial activity of amphipathic molecules^12^. Therefore, tuning the amphiphilic properties through modifying the molecular dimensions and lipophilicity that used to break through the biophysical barrier, which should have a critical impact on the biological activities of the modified molecules. 2,4-DAPG is an amphipathic molecule with special diacetyl groups, which enable structural modification resulting in profound molecular diversity that could be readily achieved with chemical synthetic approaches^13^. Accordingly, it is feasible approach to design a series of 2, 4-DAPG analogues by replacing its acetyl groups with substitutes in different lengths and dimensions to modulate the amphiphilic properties.

In the present study, fourteen 2, 4-DAPG analogous were chemically synthesized and their structure-activity relationships (SAR) were further evaluated against the *P. digitatum* and *P. italicum*. Among the analogous, it was found that **MP4** showed the highest antifungal activities against those two aforementioned pathogens. In addition, a possible action mechanism of **MP4** against the pathogens was proposed. Finally, its toxicity was evaluated in compliance with the relevant regulations.

## Results

### Syntheses of 2,4-DAPG analogous

**Figure 1:**
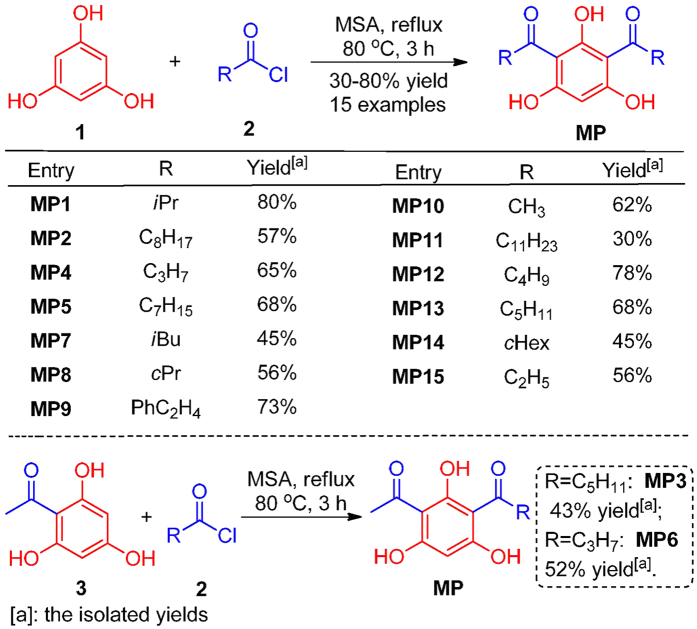
Synthesis of 2, 4-DAPG analogous. The yield was calculated as the ratio of isolated quantities to theoretical quantities.

The commercially available phloroglucinol **1** and acyl chloride **2** were used as the starting materials, which were exposed to the neat MSA (Methanesulfonic acid) solution and heated at 80 °C for 3 h, resulting in the desirable products **MP1-2**, **MP4-5** and **MP7**–**15** in moderate to excellent efficiency (52–84% yield) (Fig. 1). Notably, the preparation of the unsymmetrical 2,4-DAPG analogous **MP3** and **MP6** was carried out using the readily available acetylphloroglucinol **3** and acyl chloride **2,** resulting in the yields of 43% and 52%, respectively (Fig. 1).

### Antifungal activities of the 2, 4-DAPG analogous

**Figure 2:**
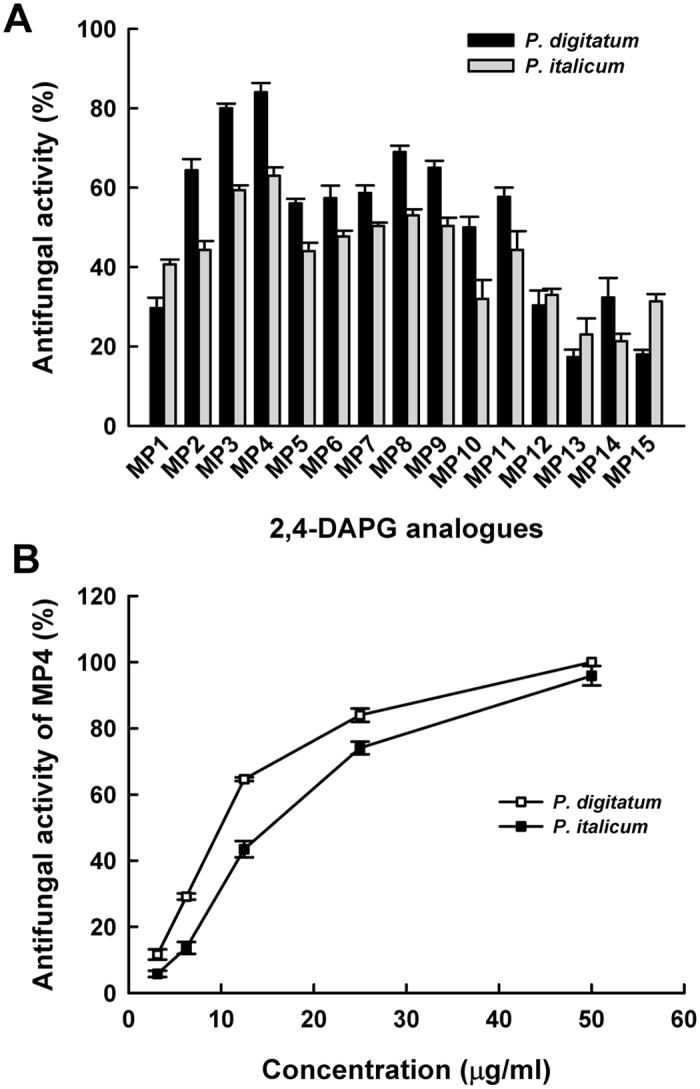
(**A**) *In vitro* antifungal activities of 2, 4-DAPG analogues against *P. italicum* and *P. digitatum* at the concentration of 25 μg/mL. (**B**) *In vivo* antifungal activities of **MP4** against *P. italicum* and *P. digitatum* at different concentrations.

**Figure 3:**
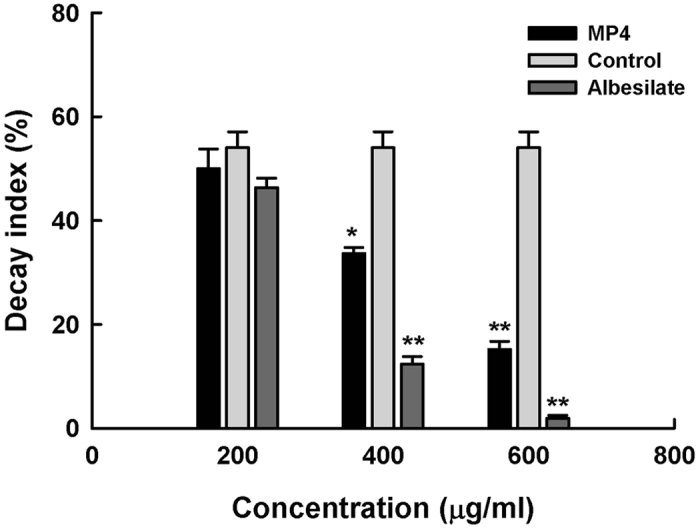
*In vivo* effectiveness evaluation of MP4 used for post-harvest sugar orange in comparison with albesilate at the concentrations of 200, 400 and 600 μg/mL. Statistical analysis was performed using SigmaPlot software, version 8.0 (mean ± standard error, with *p < 0.05 and **p < 0.001).

The inhibition ratio of 2, 4-DAPG (**MP1**) at the concentration of 25 μg/mL against the *P. digitatum* and *P. italicum* were 29.7% and 40.7%, respectively. In comparison, most of the synthesized 2,4-DAPG analogues had exhibited higher antifungal activities than 2, 4-DAPG (Fig. 2). Among these analogues, at the concentration of 25 μg/mL, **MP4** showed the antifungal activities of 84.0% and 63.0% against *P. digitatum* and *P. italicum*, respectively, while the values were 80.0% and 59.3% for **MP3**, respectively (Fig. 2A). In addition, the fungicidal activity of **MP4** was shown with a concentration-dependent manner, from which it was estimated the EC_50_ values of **MP4** against the *P. digitatum* and *P. italicum* were 8.2 (R^2^ = 0.99) and 14.4 (R^2^ = 0.98) μg/ml, respectively (Fig. 2B). Under the natural infected conditions, decay index for control mandarin (*Citrus reticulata* Blanco cv. Shatang.) fruit was 54.0% after 20 days of storage. Application of MP4 at the concentrations of 400 μg/mL or more significantly inhibited citrus fruit decay. When the **MP4** was used at 600 μg/mL, the decay index was decreased to 15.1%, which implied **MP4** to possess a competitive efficiency compared to the positive control, albesilate (12.3%) at 400 μg/mL (Fig. 3).

### Effects of MP4 on the mycelial morphology and cytochrome p450 (*CYP*) gene expression

**Figure 4:**
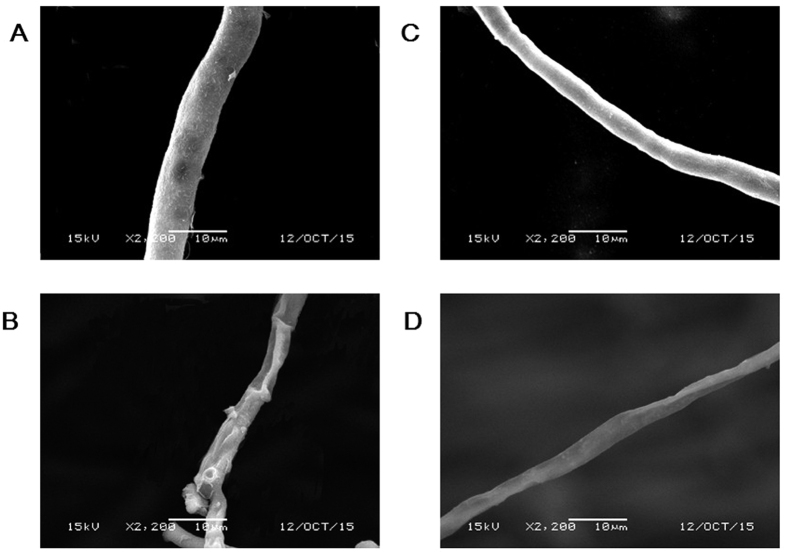
Scanning electron micrographs of hyphae of *P. digitatum* and *P. italicum* grown for 5 days on PDA medium containing MP4 at 25 °C. (**A**) *P. digitatum*, control; (**B**) *P. digitatum*, MP4; (**C**) *P. italicum*, control; (**D**) *P. italicum*, MP4. Scale bars = 10 μm.

**Figure 5:**
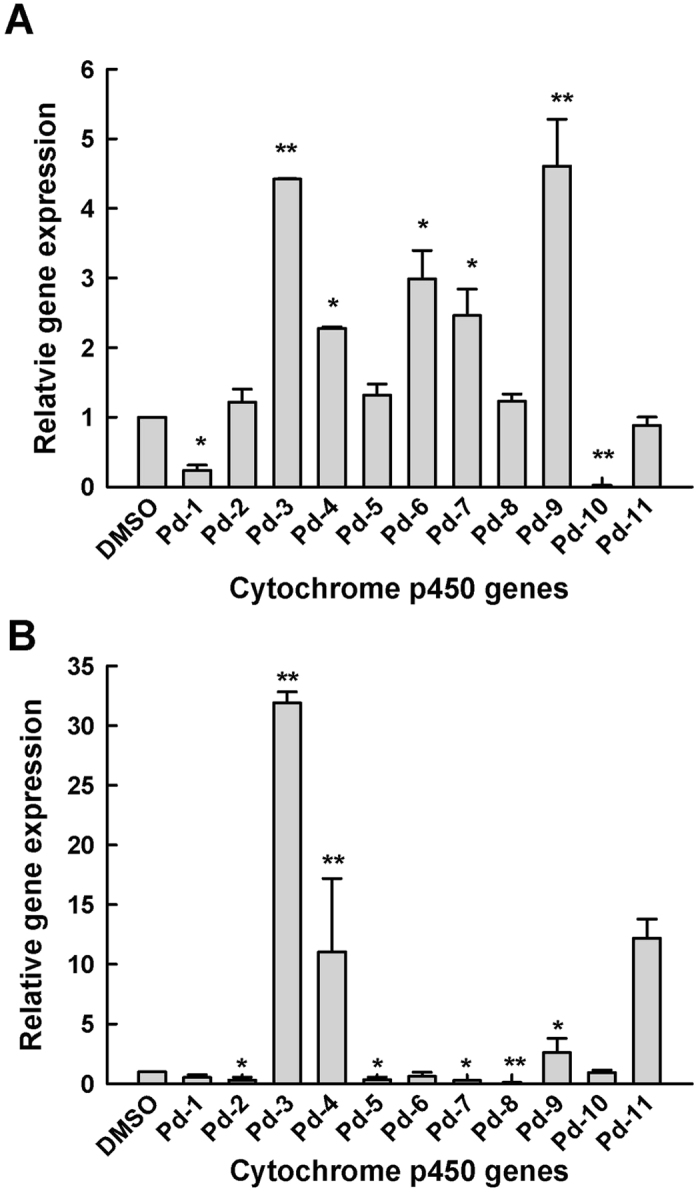
Expression profile of cytochrome P450 genes in mycelia of *P. digitatum* (**A**) and *P. italicum* (**B**) grown for 5 days on PDA medium containing MP4 at 25 °C. Statistical analysis was performed using SigmaPlot software, version 8.0 (mean ± standard error, with *p < 0.05 and **p < 0.001).

The damages caused by **MP4** on mycelial morphology of both *P. digitatum* and *P. italicum* were investigated by SEM. The results showed that application of MP4 resulted in the wrinkle of the hyphae in both fungi with serious folds and breakage (Fig. 4). In addition, the expression of *CYP* genes in both *P. digitatum* and *P. italicum* were also modified by **MP4** treatment (Fig. 5). In the case of *P. digitatum*, five *CYP* genes were significantly up-regulated, of which the expression of *Pd-9* and *Pd-3* genes were increased by 5.3-fold and 4.4-fold, respectively. On the contrary, two *CYP* genes were significantly down-regulated, of which *Pd-10* gene decreased by 43.5-fold. Nevertheless, 4 of 11 *CYP* genes were not significantly changed (Fig. 5A). Similarly, four *CYP* genes in the *P. italicum* were significantly up-regulated, of which *Pi-3* and *Pi-4* genes were increased by 32.8 and 17.2 folds, respectively. Four *CYP* genes were significantly down-regulated, of which *Pi-8* decreased by 11.1-fold. However, 3 of *CYP* genes were not significantly changed (Fig. 5B).

### Toxicology of MP4

**Figure 6:**
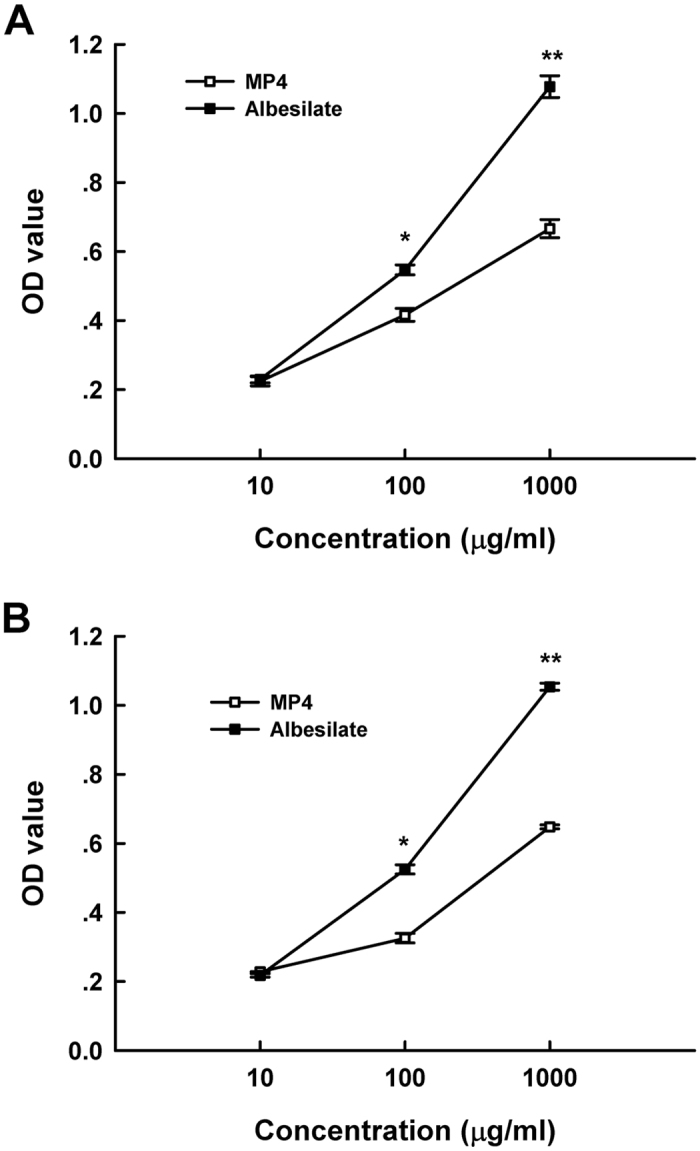
Cytotoxicity tests of **MP4** compared to albesilate using human HEK293 (**A**) and 16HEB (**B**) cell lines. Statistical analysis was performed using SigmaPlot software, version 8.0 (mean ± standard error, with *p < 0.05 and **p < 0.001).

The toxicity of **MP4** was preliminarily evaluated by detecting the proliferation of the 16HBE and HEK293 cells subjected to the treatment of **MP4** and the positive control drug, albesilate. As shown in Fig. 6, both **MP4** and albesilate inhibited the proliferation of the 16HBE and HEK293 cells in a dose–response pattern. However, the toxicity of **MP4** was significantly lower than that of albesilate (Fig. 6). At the high concentration of 1000 μg/mL, **MP4** inhibited the cell proliferation of 16HBE and HEK293 by 64.8% and 66.6%, respectively, while the corresponding values by the positive control approached up to 100%. When the concentration of 100 μg/mL was applied, **MP4** resulted in the cell inhibitions of 16HBE and HEK293 by 32.6% and 25.3.6%, respectively. In contrast, the positive control showed the toxicity against the above mentioned cells up to 52.5% and 54.7%.

## Discussion

*P. digitatum* and *P. italicum* can cause serious damages in the postharvest citrus fruit, resulting in severe economic loss. In addition, the development of fungicidal resistance in *P. digitatum* and *P. italicum* has threatened the effectiveness of application of current fungicides. Therefore, this study seems particularly important and useful because it is the first report on the structural modification of 2, 4-DAPG to achieve higher antifungal activities against the *P. digitatum* and *P. italicum*.

2, 4-DAPG, a natural phenolic compound, has been demonstrated as an antifungal metabolite produced by some species of *Pseudomonas*. It has been known as an excellent bio-control agent to suppress the take-all, a serious soil borne disease of wheat all over the world^14^,^15^. In this study, some novel 2, 4-DAPG analogues were intentionally chemically synthesized. It is well accepted that, if the hydrophobic portion of a molecule enters into the lipid bilayer of cell membrane, it can result in disorder in the fluid bilayer^16^,^17^. However, it is still unclear whether increasing the volume of the hydrophobicity portion of phloroglucinol through synthetic modification could enhance antifungal activities. Satisfactorily, the results from antifungal screening demonstrated a clear structure-activity relationship. When more bulky lipophilic acetyl substitutes were introduced into the diacylphloroglucinol analogues (**MP1**–**MP4**), their antifungal activities dramatically increased (Fig. 2A and Supplemental 1). In the present study, **MP4** was synthesized with two butyl substituents in the phloroglucinol ring, which has exerted the most potent antifungal activities with the inhibition rates of 84% and 63% against *P. digitatum* and *P. italicum*, respectively. In contrast, the 2,4-DAPG only had the antifungal activities of 29.7% and 40.7%, respectively. However, it is worthwhile to note that some other 2,4-DAPG analogues which possess even longer alkyl tails or branching or cyclic substitutes such as **MP5**–**MP15** showed decreased antifungal activities.

Generally speaking, the analogues’ antifungal activities do not linearly correlate with their lipophilicities, although the lipophilicity seems to be a principal factor to influence the antibacterial activity. This property is certainly correlated with the ability of a compound to be diffused into biological membranes to reach its site of action. Usually, molecules with small dimensions can easily diffuse across the membranes, but they may lack the ability to disorder the fluid bilayer. However, the alkyl tails with excessively-long chains also hamper (**MP13**–**MP15**) the antifungal activity due to their poor compatibility with the lipid bilayer of cell membrane. Our results suggested that the desirable structure feature of the diacetylphloroglucinol derivatives should have a suitable lipophilicity with diacetyl substitutes of chain length between 6–8 carbons (**MP3**–**MP4**).

CCK-8 is a sensitive colorimetric assay, which is often used as a common way to determine the toxicity levels of pesticides before the chemicals are launched into market^18^. As shown in Fig. 6, the cytotoxicity of **MP4** was significantly lower than that of albesilate, a widely used fungicide in citrus. Currently, health risk and environmental pollution issues associated with fungicides and pesticides have become increasingly prominent. Thiabendazole is another widely utilized fungicide in post-harvest citrus. It has been reported that thiabendazole was a potential hazard for many prokaryotic and eukaryotic organisms, especially with genotoxic effect on human peripheral lymphocytes^19^. The adverse effects of other post-harvest fungicides, such as prochloraz and imazalil, were also reported to inhibit secretion of cortisol and aldosterone in human adrenocortical H295R cells^20^. Our results suggested that **MP4** might provide a safe alternative to fungicide against *P. digitatum* and *P. italicum*.

One of the action mechanisms of fungicides, such astricyclazole, was reported to exert the influence on fungal cell membrane formation by inhibiting the expression of cytochrome P450 14α-demethylase (CYP51)^21^, which is a very important enzyme responsible for the biosynthesis of ergosterol^22^. In the present study, **MP4** resulted in the significant inhibition of hypha growth in *P. digitatum* and *P. italicum*. Also the surfaces of hyphae were wrinkled with folds and breakage, which indicated that the cell membrane formation was affected. Furthermore, 2 and 4 *CYP* genes were significantly down-regulated by **MP4** in *P. digitatum* and *P. italicum*, respectively. These results suggested a possible action mechanism of **MP4** against the *P. digitatum* and *P. italicum*, which might rely on the damage upon the fungal cell membrane and this process may be associated with the down-regulation of some *CYP* genes in these two pathogens.

In regard to the inducible expression of *CYP* genes, it may not be a clear implication to directly explain the action mechanism of **MP4** for its control of *P. digitatum* and *P. italicum*, but it demonstrated that **MP4** could induce the defense responses of these two pathogens. As frequently reported, fungal *CYP* genes could be induced in the response to environmental stresses. For examples, the heavy metals, such as cadmium, increased the expression levels of cytochrome P450 in *Phanerochaete chrysosporium*^23^; similarly, cytochrome P450 genes could be induced by high and low temperatures, osmotic stress, oxidative stress etc. in a wood decaying fungi *Heterobasidion annosum*^24^. Therefore, it seems that the up-regulated P450 genes in *P. digitatum* and *P. italicum*were in relationship to the stress tolerance of these two pathogens underlying the treatment of **MP4**. Although the accurate mechanism of action of MP4 against the *P. digitatum* and *P. italicum* remain uncertain and needs to be further studied, our study has provided, in a certain extent, some useful information about **MP4** that could impact effects on the mycelial morphology and the expression of P450 genes in the *P. digitatum* and *P. italicum*.

## Methods

### Materials and experimental apparatus

The syntheses of the 2,4-DAPG analogues were conducted with MSA-catalyzed Friedel-Crafts acylation according to the previously reported procedures^25^ with slight modification as outlined in Fig. 1. All reactions were carried out under an air atmosphere with dry solvents unless otherwise noted. All the reagents were purchased at highly commercial quality and used without further purification. Thin-layer chromatography was conducted with 0.25 mm Tsingdao silica gel plates (60F-254) and visualized by exposure to UV light (254 nm) or stained with potassium permanganate. Silica gel (200–300 mesh) used for flash column chromatography was purchased from Qing Dao Hai Yang Chemical Industry Co. in China. ^1^H NMR and ^13^C NMR spectra were recorded on a Brüker Advance 500 (^1^H: 500 MHz, ^13^C: 125 MHz). Chemical shifts reported in parts per million relative to CDCl_3_ (^1^H NMR; 7.27 ppm, ^13^C NMR; 77.00 ppm) and CD_3_OD (^1^H NMR; 3.33 ppm, ^13^C NMR; 47.60 ppm). Yields referred to chromatographically purified products unless otherwise stated. The following abbreviations were used to explain the multiplicities: s = singlet, d = doublet, t = triplet, q = quartet, m = multiplet, br = broad.

### General procedure for the syntheses of 2,4-DAPG analogous MP1-2, MP4-5 and MP7-15

A flame-dried 10 mL flask was charged with phloroglucinol**1** (2.5 mmol, 315 mg) and acyl chloride **2** (5.0 mmol) before MSA (25 mmol, 1.6 mL) was added. The resulting mixture was allowed to be stirred at 80 °C for 3 h until most of the starting materials were consumed prior to the TLC detection. Then, the mixture was poured into a mixture of water (25 mL) and EtOAc (25 mL). The organic phase was separated and the aqueous phase was well extracted with EtOAc (3 × 25 mL). Then, the organic phases were combined and washed with water (2 × 25 mL) and brine (25 mL), dried over Na_2_SO_4_ and concentrated in vacuum to give a crude solid. The crude product was further purified with a short flash column chromatography (silica gel, hexane/EtOAc = 5:1) to afford the corresponding products. Characterization spectral data of the synthetic compounds were provided in the Supplemental file 1.

### General procedure for the syntheses of 2,4-DAPG analogues MP3 and MP6

A flame-dried 10 mL flask was charged with acylphloroglucinol **3** (2.0 mmol, 336 mg) and acyl chloride **2** (3.0 mmol) before the MSA (15 mmol, 1.0 mL) was added. The remaining procedures to obtain MP3 and MP6 were performed as the above-mentioned. Characterization of spectral data of the synthetic compounds was provided in the Supplemental file 1.

### *In vitro* assay

There were two rounds of activity screening for the synthesized 2, 4-DAPG analogues. At first, each synthesized 2, 4-DAPG analogues was dissolved in dimethyl sulfoxide (DMSO) at a concentration of 1000 μg/mL, then they were diluted to the final concentration of 25 μg/mLin the PDA (potato dextrose agar) medium, mixed well, then poured into 9 cm culture dishes. After the media in the dishes were cooled down and solidified, the pathogens including *P. digitatum* and *P. italicum* (kindly provided by professor. MY Hu, College of Agriculture, South China Agricultural University, Guangzhou, China) were inoculated separately on the surface of the media using an assay disc (0.4 cm in diameter) cut. At second, based on the first round of screening, the most efficient 2, 4-DAPG analogue (i.e., **MP4**) was selected to be used in subsequent experiments. The **MP4** solution was diluted to a concentration gradient of 50, 25, 12.5, 6.25, 3.125 μg/mL in the PDA medium, respectively. The assay was conducted with the procedures as the above-mentioned, while equal portion of DMSO was used as a negative control. All the incubations were kept in 25 °C for 5 days. The percent rate of inhibition was calculated based on the following formula:


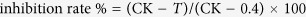


where CK is the colony diameter (cm) of the negative control sample, and *T* is the colony diameter (cm) of the samples treated as above. All experiments were carried out with three experimental replicates and the concentrations of the EC_50_ (half maximal effective concentration) for **MP4** against the *P. italicum* and *P. digitatum* were analyzed using the GraphPad Prism software, version 5.01 (Graphpad Software, Inc.).

### *In vivo* assay

Naturally infected mandarin (*Citrus reticulata* Blanco cv. Shatang.) fruit were selected with uniform size and no visible mechanical wounds, and used to test the *in vivo* biological activities of **MP4.** Distilled water and albesilate instead of **MP4** were regarded as negative and positive control, respectively. MP4 was diluted to the concentration gradient of 200, 400, 600 μg/mL in 1% tween 80 solution. mandarin fruit were soaked in the corresponding solutions for 5 min, respectively. After natural drying, the treated fruit were kept in an air-conditioning room (25 °C) for 20 days. The decay extent of mandarin fruit were recorded by the following scores based on an organoleptic test, as follows: 1 = no decay, 2 = slight decay, 3 = 25–50% decay, and 4 > 50% decay. The decay index was further calculated as reported (Liang *et al.*^27^ R = (decay scale × proportion of fruit corresponding to each scale)/5n × 100% (where n = the number of fruit investigated). Each treatment consisted of three replicates, with 60 fruit per replicate.

### Real-Time PCR detection

*P. digitatum* and *P. italicum* were cultured in the PDA fluid medium under the exposure of 7 μg/ml and 16 μg/mLof **MP4** after 5 days, respectively. These two values were the EC_50_ values of MP4 against the *P. Digitatum* and *P. italicum,* respectively. Equal part of DMSO was used as a negative control. An amount of 100 mg of mycelia were collected and used for the RNA isolation using HiPure Fungal RNA Mini Kit (Magen, Guangzhou, China). Then the cDNA was synthesized by the PrimeScriptTM RT Master Mix (TAKARA, Dalian, China). Cytochrome P450 (*CYP*)genes of the *P. digitatum* and *P. italicum* were obtained from the genome sequencing of these two species (More details are described in the Supplemental file 2). The primer sets were designed according to the nucleotide sequence of each cytochrome P450 gene and were subjected to the RT-PCR amplification in a 7500 Fast Real-Time PCR System (Applied Biosystems, CA, USA) with a 20 μLreaction system including 10 μLSYBR Premix Ex TaqTM (TaKaRa, Dalian, China), 0.4 μLPCR forward primer (10 mM), 0.4 μLPCR reverse primer (10 mM), 0.4 μLROX reference dye II, 2 μL(20 ng) cDNA and 6.8 μLddH_2_O. Amplification conditions were as the following: 30 s at 95 °C, followed by 40 cycles at 95 °C for 5 s, 60 °C for 34 s. Each sample was performed with three independent biological replicates and each biological test was with three technical replicates. After the reaction was performed, all dissociation curves showed a single amplification peak for each reaction. The relative expressions were first normalized to the endogenous reference gene, and then normalized to the gene expression level in the DMSO-treated samples according to the 2^−△△Ct^ method^26^.

### Scanning Electron Microscopy

The surface morphology of hyphae of *P. digitatum* and *P. italicum* were observed by a scanning electron microscopy (SEM) according to the method described by Liang *et al.*^27^.

### Cell proliferation assay

Human bronchial epithelial (16HBE) cells and human embryonic kidney 293 cells (HEK293) were obtained from American Type Culture Collection (ATCC) and cultured in the DMEM medium and 1640 RPMI medium, respectively, supplemented with 10% fetal bovine serum (FBS) and 1% penicillin or streptomycin. The cells were incubated at 37 °C in a humidified atmosphere containing 5% CO_2_, and harvested prior to the use for the subsequence experiments.

Cell proliferation assay was performed to investigate the toxicity of **MP4** using cell counting kit-8 (Dojindo Laboratories, Kumamoto, Japan). Albesilate (generously provided by Dr. Ehrenstorfer GmbH, Augsburg, Germany) was used as a positive control. All the experimental procedures were performed according to the manufacturer’s introduction. Briefly, the cells were sub-cultured in 96-well plates at the density of 1 × 10^3^ cells/well. After the conventional incubation for 24 h, 10 μL **MP4** and albesilate solutions were added to each well, respectively, with three different concentrations (i.e., 1000, 100, 10 μg/mL). An aliquot of 10 μLDMSO was used as a negative control. After the conventional incubation for 12 h, 10 μL CCK-8 was added to each well. Followed by incubation for 3 h at 37 °C, the OD values were detected at 450 nm (A450) using a microplate spectrophotometer (Bio-RAD instruments, CA, USA). All experiments were carried out with three biological replicates and each biological test was with three technical replicates.

### Statistical analysis

Statistical analysis was performed with the GraphPad Prism software, version 5.01 (Graphpad Software, Inc.) using analysis of variance, followed by multiple comparisons with the one way ANOVA. P < 0.05 was considered as with statistically significant difference.

## Additional Information

**How to cite this article**: Gong, L. *et al.* Novel synthesized 2, 4-DAPG analogues: antifungal activity, mechanism and toxicology. *Sci. Rep.* **6**, 32266; doi: 10.1038/srep32266 (2016).

## Supplementary Material

Supplementary Information
