# Supplementary material for: Novel synthesized 2, 4-DAPG analogues: antifungal activity, mechanism and toxicology
Source: Sci Rep. 2016 Aug 26;6:32266. doi: 10.1038/srep32266 (PMC4999805; doi:10.1038/srep32266)
Supplement: Supplementary Information [file srep32266-s1.pdf]

Novel synthesized 2, 4-DAPG analogues: antifungal activity, mechanism and toxicology

Liang Gong[1#], Haibo Tan[1#], Feng Chen[2], Taotao Li[1], Jianyu Zhu[3], Qijie Jian[1], Debao, Yuan[1], Liangxiong Xu[1], Wenzhong Hu[4], Yueming Jiang[1], XuewuDuan[1,*]

Additional information

Supplemental file 1: NMR spectroscopy information on **MP1-MP15**

Supplemental file 2: Primers used for qRT-PCR

Supplemental file 1: NMR spectroscopy information on MP1-MP15

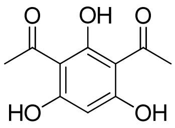

MP1

1,1'-(2,4,6-trihydroxy-1,3-phenylene)diethanone MP1

^{1}H NMR (500 MHz, CD_3OH+$CDCl_3$): δ = 5.75 (s, 1H), 2.60 (s, 6H); ^{13}C NMR (125 MHz, $CDCl_3$): δ = 203.2, 170.6, 168.2, 103.0, 93.8, 31.3.

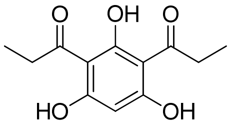

MP2

1,1'-(2,4,6-trihydroxy-1,3-phenylene)bis(propan-1-one) MP2

^{1}H NMR (500 MHz, $CDCl_3$): δ = 5.83 (s, 1H), 3.16 (q, J = 7.2 Hz, 3H), 1.20 (t, J = 7.2 Hz, 5H); ^{13}C NMR (125 MHz, $CDCl_3$): δ = 171.8, 95.3, 37.4, 31.0, 8.5.

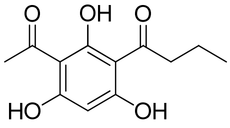

MP3

1-(3-acetyl-2,4,6-trihydroxyphenyl)hexan-1-one MP3

^{1}H NMR (500 MHz, $CDCl_3$): δ = 5.83 (d, J = 3.4 Hz, 1H), 3.10 (t, J = 7.2 Hz, 2H), 2.72 (s, 3H), 1.72 (q, J = 7.4 Hz, 2H), 1.02 (t, J = 7.4 Hz, 3H); ^{13}C NMR (125 MHz, $CDCl_3$): δ =204.2, 172.0, 95.2, 46.0, 32.8, 18.0, 13.9.

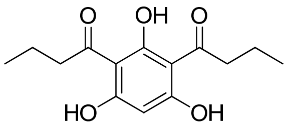

MP4

1,1'-(2,4,6-trihydroxy-1,3-phenylene)bis(butan-1-one) MP4

^{1}H NMR (500 MHz, $CDCl_3$): δ = 5.87 (s, 1H), 3.08 (t, J = 9.2 Hz, 4H), 1.73 (3, 4H), 1.01 (t, J = 7.4 Hz, 6H); ^{13}C NMR (125 MHz, $CDCl_3$): δ = 207.1, 172.0, 104.1, 95.3, 46.0, 18.0, 13.9.

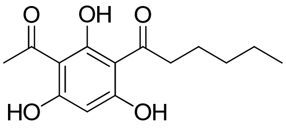

MP5

1-(3-acetyl-2,4,6-trihydroxyphenyl)hexan-1-one MP5

^{1}H NMR (500 MHz, $CDCl_3$): δ = 5.86 (s, 1H), 3.10 (t, J = 7.5 Hz, 2H), 2.72 (s, 3H), 1.70 (m, 2H), 1.37 (m, 4 H), 0.99 (m, 3H); ^{13}C NMR (125 MHz, $CDCl_3$): δ = 207.3, 204.4, 172.1, 104.1, 95.3, 44.1, 32.8, 31.6, 24.3, 22.6, 14.0.

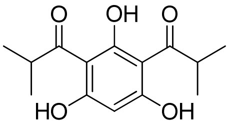

MP6

1,1'-(2,4,6-trihydroxy-1,3-phenylene)bis(2-methylpropan-1-one) MP6

^{1}H NMR (500 MHz, $CDCl_3$): δ = 5.87 (s, 1 H), 3.96 (t, J = 6.7 Hz, 1H), 1.21 (d, J = 6.7 Hz, 1H); ^{13}C NMR (125 MHz, $CDCl_3$): δ = 211.5, 182.4, 172.2, 103.6, 95.6, 39.3, 19.2.

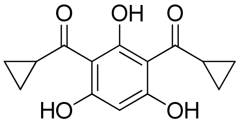

MP7

(2,4,6-trihydroxy-1,3-phenylene)bis(cyclopropylmethanone) MP7

^{1}H NMR (500 MHz, $CDCl_3$): δ = 5.78 (s, 1H), 4.17 (s, 2H), 2.0-2.5 (m, 8H); ^{13}C NMR (125 MHz, $CDCl_3$): δ = 169.6, 95.3, 45.0, 24.7, 21.2.

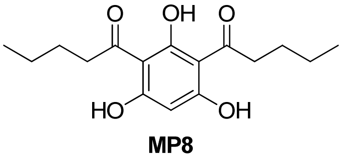

MP8

1,1'-(2,4,6-trihydroxy-1,3-phenylene)bis(pentan-1-one) MP8

^{1}H NMR (500 MHz, $CDCl_3$): δ = 5.83 (s, 1H), 3.11 (t, J = 7.5 Hz, 4H), 1.69 (dt, J = 15.0, 7.5 Hz, 4H), 1.42 (dd, J = 15.0, 7.5 Hz, 4H), 0.97 (t, J = 7.5 Hz, 6H). ^{13}C NMR (125 MHz, $CDCl_3$): δ = 207.3, 172.0, 95.4, 43.9, 26.8, 24.5, 14.0.

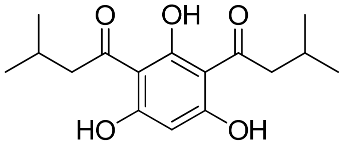

MP9

1,1'-(2,4,6-trihydroxy-1,3-phenylene)bis(3-methylbutan-1-one) MP9

^{1}H NMR (500 MHz, $CDCl_3$): δ = 5.87 (s, 1H), 2.99 (d, J = 6.7 Hz, 4H), 2.27 (m, 2H), 1.00 (d, J = 6.7 Hz, 6H); ^{13}C NMR (125 MHz, $CDCl_3$): δ = 206.8, 179.2, 172.1, 104.3, 95.4, 43.1, 25.6, 22.8.

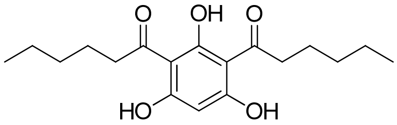

MP10

1,1'-(2,4,6-trihydroxy-1,3-phenylene)bis(hexan-1-one) MP10

^{1}H NMR (500 MHz, $CDCl_3$): δ = 5.83 (s, 1H), 3.10 (t, J = 7.4 Hz, 4H), 1.71 (m, 4H), 1.37 (m, 8H), 0.93 (m, 6H); ^{13}C NMR (125 MHz, $CDCl_3$): δ = 171.9, 95.4, 44.1, 31.6, 24.4, 22.6, 14.0.

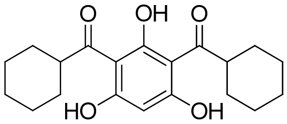

MP11

(2,4,6-trihydroxy-1,3-phenylene)bis(cyclohexylmethanone) MP11

^{1}H NMR (500 MHz, $CDCl_3$): δ = 5.79 (s, 1H), 4.44 (s, 4H); ^{13}C NMR (125 MHz, $CDCl_3$): δ = 204.1, 172.1, 134.7, 129.9, 128.5, 126.9, 95.7, 50.1.

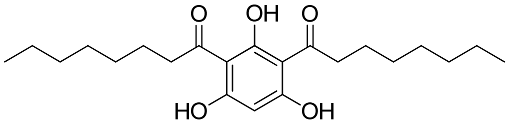

MP12

1,1'-(2,4,6-trihydroxy-1,3-phenylene)bis(octan-1-one) MP12

^{1}H NMR (500 MHz, $CDCl_3$): δ = 5.83 (s, 1H), 3.11 (t, J = 7.5 Hz, 4H), 1.69 (dt, J = 15.0, 7.5 Hz, 4H), 1.31 (m, 16H), 0.90 (t, J = 7.5 Hz, 6H). ^{13}C NMR (125 MHz,

$CDCl_3$): δ = 207.3, 172.0, 95.4, 44.5, 31.8, 29.5, 29.2, 29.1, 25.1, 14.1.

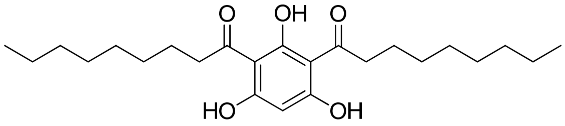

MP13

1,1'-(2,4,6-trihydroxy-1,3-phenylene)bis(octan-1-one) MP13

^{1}H NMR (500 MHz, CDCl3): δ = 5.83 (s, 1H), 3.11 (t, J = 7.5 Hz, 4H), 1.69 (dt, J = 15.0, 7.5 Hz, 4H), 1.31 (m, 20H), 0.90 (t, J = 7.5 Hz, 6H). ^{13}C NMR (125 MHz, $CDCl_3$): δ = 207.3, 172.0, 95.4, 44.3, 31.8, 29.6, 29.5, 29.2, 29.1, 25.0, 14.1.

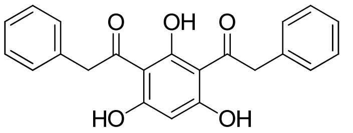

MP14

1,1'-(2,4,6-trihydroxy-1,3-phenylene)bis(2-phenylethanone) MP14

^{1}H NMR (500 MHz, $CDCl_3$): δ = 7.20-7.40 (m, 10H), 5.79 (s, 1H), 4.44 (s, 4H); ^{13}C NMR (125 MHz, $CDCl_3$): δ = 204.1, 172.1, 134.7, 129.9, 128.5, 126.9, 95.7, 50.1.

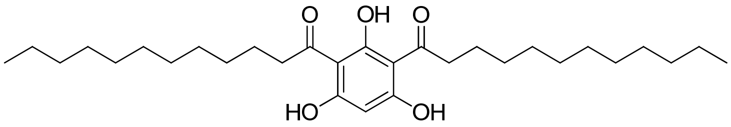

MP15

1,1'-(2,4,6-trihydroxy-1,3-phenylene)bis(dodecan-1-one) MP15

^{1}H NMR (500 MHz, $CDCl_3$): δ = 5.81 (s, 1H), 3.10 (t, J = 7.4 Hz, 4H), 1.70 (m, 4H), 1.31 (m, 32H), 0.90 (t, J = 7.5 Hz, 6H). ^{13}C NMR (125 MHz, $CDCl_3$): δ = 171.9, 95.4, 44.2, 31.9, 29.6, 29.6, 29.5, 29.3, 29.2, 29.1, 24.7, 22.7, 14.1.

Supplemental file 2: Primers used for qRT-PCR

Sequence Name	Sense Primer(5'-3')	Anti-sense Primer (5'-3')	GenBank No.
**DP1**	CGCCTTCAACCTCCTGTGCCTATCA	AACGACGGTGTAGCTTGCCTCAGT	AKCT01000203.1
**DP2**	CAGACCGCTTCTTCTACGAAGGTATGGA	GTCAGAGCCTCGGCAGTCAGTTCA	AKCT01000285.1
**DP3**	AGTCTTCCAGCAATACCTCCCTAGTCTACA	CCGCCGCAGCAAGATACTCATCAATG	AKCT01000203.1
**DP4**	CAACCATCCTCTTCCACCTCATTCACAAC	TGTAAGTTATACGCAGCACAGCCTACGA	AKCT01000102.1
**DP5**	CCTGATGCGTTGCGTGTATCGTGAC	CATAAGCGTGTGAATGGAAGAGTGGAGAC	AKCT01000102.1
**DP6**	TCCTGATGACGCCGCAGAAGATGAA	AGTGATTAACCGAGAGCACAGTCTTTCCT	AKCT01000328.1
**DP7**	AGTGAACTGAAACGGTCAACTATGCCATTT	TCTCAGCAGCGTTACAACAGACAGGAA	AKCT01000010.1
**DP8**	GACTACCAAGGATGAAGATGAGGAGCAGAT	ACCAGCACCGAAAGGCAGATAAGGA	AKCT01000064.1
**DP9**	CCTGAATCTGAGTACCATCCTGACCATACC	CCAGTCCAGCCGCAATAACTTCTTGAG	AKCT01000319.1
**DP10**	GACCTACTCGTGGCTACACACAGTGATAG	TGAACATTCTTCTCGTCCGTCTCGTGAA	AKCT01000136.1
**DP11**	TGTGGTCGCTGGTTATTCGTCTCTTCA	TAGTACCGAGTTCACCTTCAGGCAGATTG	AKCT01000232.1
**IP1**	AGCACCGCCGATACCGTCATTGA	CGTTGCTGCGAGCGTCGTCATT	JQGA01001117.1
**IP2**	ACGATCCAGCCTTCTACCACGAGTTG	TGCCAAAGACTTCAAAGCCCATTCCATC	JQGA01001283.1

**IP3**	GCGAGAACAGAGGGCAGGCTTACA	GTCCGTGACAGGCTCAGGAATACCA	JQGA01000239.1
**IP4**	AGGAGCGAGGAGATGCTGGAATTGG	TTCCCGCACCATCCCATCCATATCAA	JQGA01001611.1
**IP5**	TCTCGCACGACACCAGCACATCTT	CGCCGCCAGAAGGAAGAGTAGTGT	JQGA01000007.1
**IP6**	CATCCCACCATCTTCACAGACATCCTACA	ACCAGCTCAGAGACCAGTCGTTGTTC	JQGA01000952.1
**IP7**	GGACGAACTCAACCTCCACGGTGAT	GTGTTGCCGATGGTGATGCCTTCTG	JQGA01001488.1
**IP8**	TGACGCACACGCTATCAACATCAACAA	TAGAATACCACGGACCATCTTACCACCAAG	JQGA01000925.1
**IP9**	CTGCCAAGGAAGGAAGGTCGCTACT	TTCTCCGCCGTCTCCCACTCTTTG	JQGA01000228.1
**IP10**	GAGATTGGAGCGGAGAGCACTACTATGATT	GGTAGCGGCAGAGGTAGAAGAAGATTGAT	JQGA01001615.1
**IP11**	ACGGAGCTAGAGTGCTTGCCTTACC	ACCGCCTCATTCAACCACCGATCA	JQGA01000867.1

DP for *P. digitatum CYP* gene; IP for *P. italicum CYP gene*
